# Evoked and oscillatory EEG activity differentiates language discrimination in young monolingual and bilingual infants

**DOI:** 10.1038/s41598-018-20824-0

**Published:** 2018-02-09

**Authors:** Loreto Nacar Garcia, Carlos Guerrero-Mosquera, Marc Colomer, Nuria Sebastian-Galles

**Affiliations:** 1 Infant Studies Centre, University of British Columbia, 2136 West Mall, Vancouver, BC V6T 1Z4 Canada; 2 Center for Brain and Cognition, Universitat Pompeu Fabra, Ramon Trias Fargas, 25-27, 08005 Barcelona, Spain

## Abstract

Language discrimination is one of the core differences between bilingual and monolingual language acquisition. Here, we investigate the earliest brain specialization induced by it. Following previous research, we hypothesize that bilingual native language discrimination is a complex process involving specific processing of the prosodic properties of the speech signal. We recorded the brain activity of monolingual and bilingual 4.5-month-old infants using EEG, while listening to their native/dominant language and two foreign languages. We defined two different windows of analysis to separate discrimination and identification effects. In the early window of analysis (150–280 ms) we measured the P200 component, and in the later window of analysis we measured Theta (400–1800 ms) and Gamma (300–2800 ms) oscillations. The results point in the direction of different language discrimination strategies for bilingual and monolingual infants. While only monolingual infants show early discrimination of their native language based on familiarity, bilinguals perform a later processing which is compatible with an increase in attention to the speech signal. This is the earliest evidence found for brain specialization induced by bilingualism.

## Introduction

A crucial difference for successful language learning between infants growing up in monolingual versus bilingual environments is that bilinguals need to notice the existence of two language systems in the input, that is, they need to discriminate the languages in the environment. Previous studies have shown that although monolingual and bilingual infants show similar language discrimination abilities (i.e. bilinguals do not seem to be confused by receiving two languages), some relevant differences have been reported as well. Research with monolingual infants has shown that at birth they can discriminate some, but not all, pairs of languages. For instance, monolingual newborns can differentiate between Spanish and English^1^ or Dutch and Japanese^2^, but not between Dutch and English^3^. The fact that other mammals, such as cotton-top tamarin monkeys^4^ or Long-Evans rats^5^, can also make the distinction for very different languages (in particular Japanese and Dutch) can be taken as an indication that such differentiation abilities may be rooted in ancient evolutionary mechanisms, and therefore independent of prenatal language experience^4^.

Theoretical models of early language discriminatory abilities in humans assume that infants primarily rely on information related to prosody. One of the most popular models is the Time and Intensity Grid REpresentation proposal (TIGRE) put forth by Mehler, Dupoux, Nazzi, & Dehaene-Lambertz^6^. Their proposal suggests that, initially, infants would compute the prosodic representation of the speech input. As infants gather more experience with language and their brains mature, they would be able to integrate other characteristics of the speech stream that would allow them to make more fine-grained discriminations. Also, based on their basic timing unit, languages have being classified in three rhythmic groups as syllable-timed (i.e. Spanish or Italian); stress-timed (i.e. English or German) and mora-timed (i.e. Japanese)^7^. Abercrombie’s original classification has been subsequently challenged and modified; relevant to the research in the domain of infants’ language discrimination is the reformulation by Ramus *et al*.^8^ who proposed that language rhythm could be mapped in terms of the syllabic complexity languages allow. The TIGRE model predicts that, at birth, infants would be able to discriminate between languages of different rhythmic classes and that within-class discrimination would only be possible a few months later once their knowledge of language has increased. Experimental results have also shown the developmental pattern of language discrimination: from discrimination of languages from different rhythmic classes at birth to within-rhythmic-class discrimination starting at 4–5 months of age^1–3,9–15^. Ramus^2^ performed a series of experiments showing that newborns were able to discriminate between two languages based on rhythm alone, when distinctive phonetic information was removed. Nazzi *et al*.^14^ observed partial within-class discrimination in monolingual 5-month-old infants exposed to American English. Infants could discriminate between British and American English and between British English and Dutch, but not between Dutch and German (all of these languages belong to the same rhythmic group). The researchers concluded that the pattern of discrimination reflected infants’ refined knowledge of the prosodic properties of their native language. Converging evidence was provided by Bosch and Sebastian-Galles^9^ who showed that Spanish and Catalan could be discriminated at 4.5 months of age by Catalan or Spanish monolingual infants (experiment 2), even if sentences were low-pass filtered, therefore removing most of the phonetic information in the signal (experiment 3). Summarizing, in the first months of life, infants seem to be able to discriminate languages based on coarse rhythmic properties, and language-specific fine-grained information, such as phonemes, do not seem to start playing a relevant role until the second half of the first year of life.

A handful of studies have investigated the language discrimination abilities of bilingual infants in the first months of age. There is only one study on language discrimination abilities of bilingual newborns^16^ which shows that English monolinguals and English-Tagalog bilinguals have no difficulties in distinguishing between these two languages from different rhythmic classes at birth. A different scenario is that of infants growing up in rhythmically close languages that cannot be distinguished at birth. In this case, the earliest evidence of language discrimination refers to 4.5 month olds growing up in Spanish-Catalan bilingual homes. Spanish and Catalan are two Romance languages, rhythmically close, but different at other phonological levels (see Bosch and Sebastian-Galles^9^ for a more detailed description). Using a familiarization procedure, Bosch & Sebastian-Galles^10^ showed that 4.5-month-old Spanish or Catalan monolinguals and Spanish-Catalan bilinguals did not have any difficulty and behaved similarly when discriminating between these two languages.

While monolingual and bilingual infants seem to be equivalently capable of discriminating languages in the first six months of life, there is evidence that they may be using different mechanisms. Bosch & Sebastian-Galles^9^ reported a contrasting pattern between both groups of infants when orientation times to the native versus an unknown language were measured. Previous studies using this paradigm had shown that infants orient faster towards familiar stimuli (for auditory stimuli see Dehaene-Lambertz & Houston^17^; for visual stimuli see Schonen *et al*.^18^). Following Dehaene-Lambertz & Houston^17^, Bosch & Sebastian-Galles^9^ presented 4.5-month-old infants with sentences either in the native language (Spanish or Catalan) or a foreign language (English in Experiment 4, Italian in Experiment 5). The sentences could appear randomly at the right or the left from a central location. They measured gaze orientation latencies from the central location to the sentence source location. Monolingual infants were faster at orienting to the native language when compared with a foreign language, therefore replicating Dehaene-Lambertz & Houston’s^17^ results. However, bilingual infants showed the opposite pattern; they were slower at orienting to the native language, compared to the unknown one. The differences were restricted to the native language, as both groups showed equivalent reaction times to the foreign language. Further evidence pointing in the direction that monolingual and bilingual infants may be using different processing mechanisms in language discrimination tasks comes from studies where infants were presented with visual speech. Weikum *et al*.^19^ observed that 4- and 6-month-old monolingual infants exposed to English could discriminate between silent videos of people speaking in English and French, however at 8 months of age, they could no longer do it. But, bilingual 8-month-old infants could make the differentiation^19^, even if they have never been exposed to such languages^20^. Relevant to our work, Pons *et al*.^21^ has provided compelling evidence that at 4 months of age, bilingual infants show enhanced attention to the mouth of speakers when compared to monolinguals. These authors presented infants with videos of speakers uttering sentences in their native language (Catalan or Spanish) and a non-native one (English). They observed that bilingual infants looked more to the mouth of the speakers than monolingual ones. The authors interpreted this shift of attention as a mechanism to boost language acquisition in bilinguals. Indeed, bilinguals get on average less native language exposure (of each of their languages) than monolinguals. Therefore, they would compensate the lack of input by supplementing the auditory speech code with the redundant articulatory information provided by the mouth. Such results reflect increased processing of the speech signal at this age.

The research just reviewed shows that as early as 4 months of age there is converging evidence that monolingual and bilingual infants process connected speech in different ways. Returning to Bosch & Sebastian-Galles'^9^ results, the differences of orientation latencies could be due to different causes. Behavioral measures cannot inform about different processes taking place between the appearance of the stimulus and the infants’ response. However, electrophysiological measures of the brain activity (EEG/ERPs) can give more precise information about different intervening processes and their time-course. There are very few studies using this type of measurements to investigate speech processing in the first months of life. Additionally they have focused on studying the emergence of phonetic categories using experimental paradigms involving the repeated presentation of short stimuli^22–24^. To our knowledge, only one study run by Peña, Pittaluga and Mehler^25^ has investigated language discrimination in very young infants (3 and 6 month olds). In this study, infants were presented with sentences from different languages (native – Spanish- Italian and Japanese). The authors reported effects in two electrophysiological responses, the P200 and the Gamma band.

Peña *et al*.^25^ observed differences in the latency of the P200 component based on the age of the infants tested. The authors reported a positive component peaking around 200 ms in central-anterior bilateral electrodes. This component peaked earlier in 6-month-old infants than in 3-month-old ones. The authors considered the reduction in the P200 latency an indication of brain maturation. The P200 has been observed in other studies investigating the developmental course of the recognition of speech stimuli in newborns, 2-week-olds and 2-month olds^26,27^. Mai *et al*.^27^ tested 2 month-old infants and observed a significant larger amplitude and a trend to longer latencies in the central electrodes for a familiar voice when compared with a stranger’s one, suggesting an effect of memory and familiarity. In this study, infants heard 100 repetitions of a single word uttered by their mother and by a stranger. It is very difficult to compare the results of the just reviewed studies where the P200 has been observed because of the fundamental differences both in the age of the infants tested and in the experimental paradigms. However, it can be concluded that changes in this ERP response, either in latency or in amplitude, seem to be reliably observed when speech stimuli are presented in the first six months of age.

The study of brain oscillations, by the decomposition of the EEG signal in different frequency bands, allows for identifying different cognitive processes that take place while processing language (see Peña & Melloni^28^). To our knowledge, Peña, Pittaluga, & Mehler’s^25^ study is the only one that has reported modulations in brain oscillations in language discrimination in young infants. These authors reported increases in the Gamma band (55–75 Hz) that were exclusive for the native language in infants at 6 but not at 3 months of age, an increase that the authors related to native language discrimination. Different studies have related modulations in the Gamma and Theta bands to different aspects of speech perception. The perception of fast acoustic modulations in the speech signal (equivalent to brain activity in the Gamma Frequency 21–80 Hz) has been linked to the extraction of phonetic/segmental information, usually consonants^28–30^. Therefore, it is likely that the developmental change that Peña *et al*.^25^ observed between 3 and 6 months of age reflected the onset of the establishment of the consonant repertoire in infants, starting around six months. Using isolated syllables as stimuli, Ortiz-Mantilla *et al*.^31,32^ also reported an enhanced Gamma activity for a native syllabic contrast (unaspirated voiceless/ta/ and aspirated voiceless/ta/) as compared to a non-native one (unaspirated voiceless/ta/ and voicing-lead Spanish/da/) at 6 months of age, signaling, according to the authors, the beginning of phonological narrowing. As said, oscillations in the Theta band have also been linked to speech processing. Research on adult speech perception suggests that slow acoustic modulations of the speech stream (below 10 Hz e.g. Theta 5–8 Hz) are relevant to the extraction of syllabic and prosodic information^28^. Some studies have related increases in Theta power (5–8 Hz) to the perception of native/non-native contrast in syllables or languages both in adults and infants at six months of age^33–35^. The literature in speech perception during the first 6 months of life has shown modulations of Gamma and Theta bands in response to different native and foreign stimuli. However, no study has explored to date which band is synchronized when listening to foreign and native languages between 4 to 5 months of age.

The aim of the present investigation is to understand the differences in the mechanisms of language discrimination between monolingual and bilingual infants at 4–5 months of age. As seen, the electrophysiological evidence in early speech processing capacities is quite reduced and not always converging. An additional complication is that most of the studies have investigated brain responses to short stimuli (such as isolated syllables) with long ISIs. These stimuli facilitate the analysis of the responses, but neither mimic language in the real world, nor are suitable to study language discrimination. We adapted the experimental procedure of Peña *et al*.^25^ while keeping the procedure as similar as possible to Bosch and Sebastian-Galles^9^. We compared the neural response of infants to utterances in their native/dominant language (here either Catalan or Spanish), and two foreign unknown languages: one of the same rhythmic class (Italian) and one of a different rhythmic class (German). As 4 to 5 month-old monolingual infants begin discriminating languages of the same rhythmic class, the current design allowed us to measure the brain response to both kinds of contrasts (within and between class comparisons) in a single experiment.

Following Peña *et al*.^25^ we analyzed the processing of the speech stream in two separate windows, extracting the measures appropriate for the analyses for each of the languages and groups. In an early window (150–280 ms), we analyzed the P200 component. If, according to Peña *et al*.^25^ this component merely reflects brain maturation, we do not expect any effect, as all our infants are of the same age. However, if this component also reflects aspects of memory and familiarity, we expect to find differences as a function of language experience. In a late window, we analyzed the oscillatory activity in Theta (400–1800 ms) and Gamma band (300–2800 ms). Based in the previous review we expected the onset of those changes to take place at around 400 ms after stimulus onset^25,33^. Following Nazzi *et al*.^14^, we expect language discrimination to be related to the processing of prosodic dimensions of the native language. Based on the reviewed literature, we expect to find modulations in the Theta oscillatory activity when listening to sentences. Additionally, such differences might be stronger for the case of bilinguals. Indeed, the results with audiovisual stimuli^21^ suggest that bilinguals show enhanced processing to linguistic information, therefore, such differences might be reflected in increases in the Theta oscillatory activity. We kept the same window of analysis for Gamma as the one reported for infants in a previous study^25^. The predictions for this band of oscillations are less certain as this frequency band has been related to the processing of phonetic information in adults, and because in infants, as Peña *et al*.^25^ showed, it is unlikely to find differences between languages in such young infants before perceptual narrowing has begun.

## Results

### Event-Related Potentials, P200

#### Latency

We ran a repeated measures ANOVA with a between factor, Group (Monolingual, Bilingual) and a within factor, language (Native, Italian, German). The main effects of Group and Language did not reach significance (F < 1). The results of the ANOVA yielded a significant Group by Language interaction F(2,167) = 3.88, MSE = 0.003, p = 0.022. For each of the groups separately, we ran a one-way-ANOVA with the factor Language. The ANOVA was significant for the monolingual group F(2,81) = 4.110 MSE = 0.004, p = 0.022. No effects were significant for the bilingual group (F < 1).

**Figure 1:**
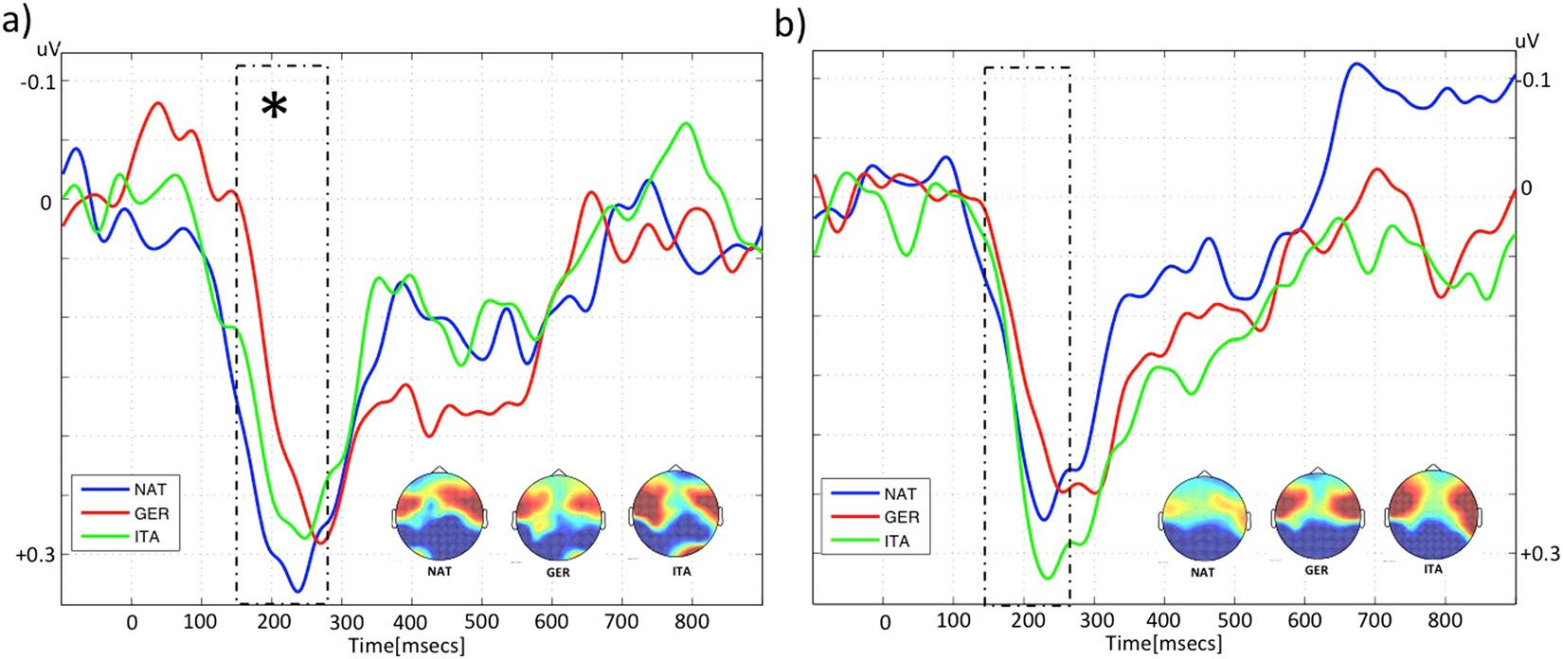
Grand-averaged event related potential waveforms of Monolingual (left) and Bilingual (right) infants to Native (blue), Italian (green) and German (red) languages. Amplitude in mv is plotted in the y axis and time in msecs is plotted in the x axis. Zero corresponds to the beggining of the utterances. ERP responses are averages over the ROI. Each arrow indicates the first substantial positive peak in the waveform which often occurs between about 150 and 275 ms. In our data, P200 component was presented between 150–280 ms (dashed box) and showed a high activation on the frontocentral area (topoplot figures).

Planned paired t-test comparisons revealed a significant difference for the Monolingual group between Native and German (t(27) = −2.891 p = 0.007) and Italian and German (t(27) = −2.501, p = 0.018) but not between Native and Italian (t < 1). This analysis confirmed that monolinguals elicited an earlier response to their native language (Mean = 232; Standard error (SE) = 0.011) than for German (Mean = 262; SE = 0.010) (See Fig. 1a). The same analysis for the bilingual group did not reach significance for any of the comparisons (see Fig. 1b).

### Time-Frequency analyses

#### Analysis in the Theta band range (4–8 Hz)

We ran a repeated measures ANOVA. The main effects of Group and Language did not reach significance (F < 1) but it yielded a significant interaction of Group by Language F(2,167) = 5.091 p = 0.009. We ran separate one-way ANOVAs for each group on the Language factor. Only the Bilingual group showed significant differences F(2,81) = 25.519 p < 0.001.

**Figure 2:**
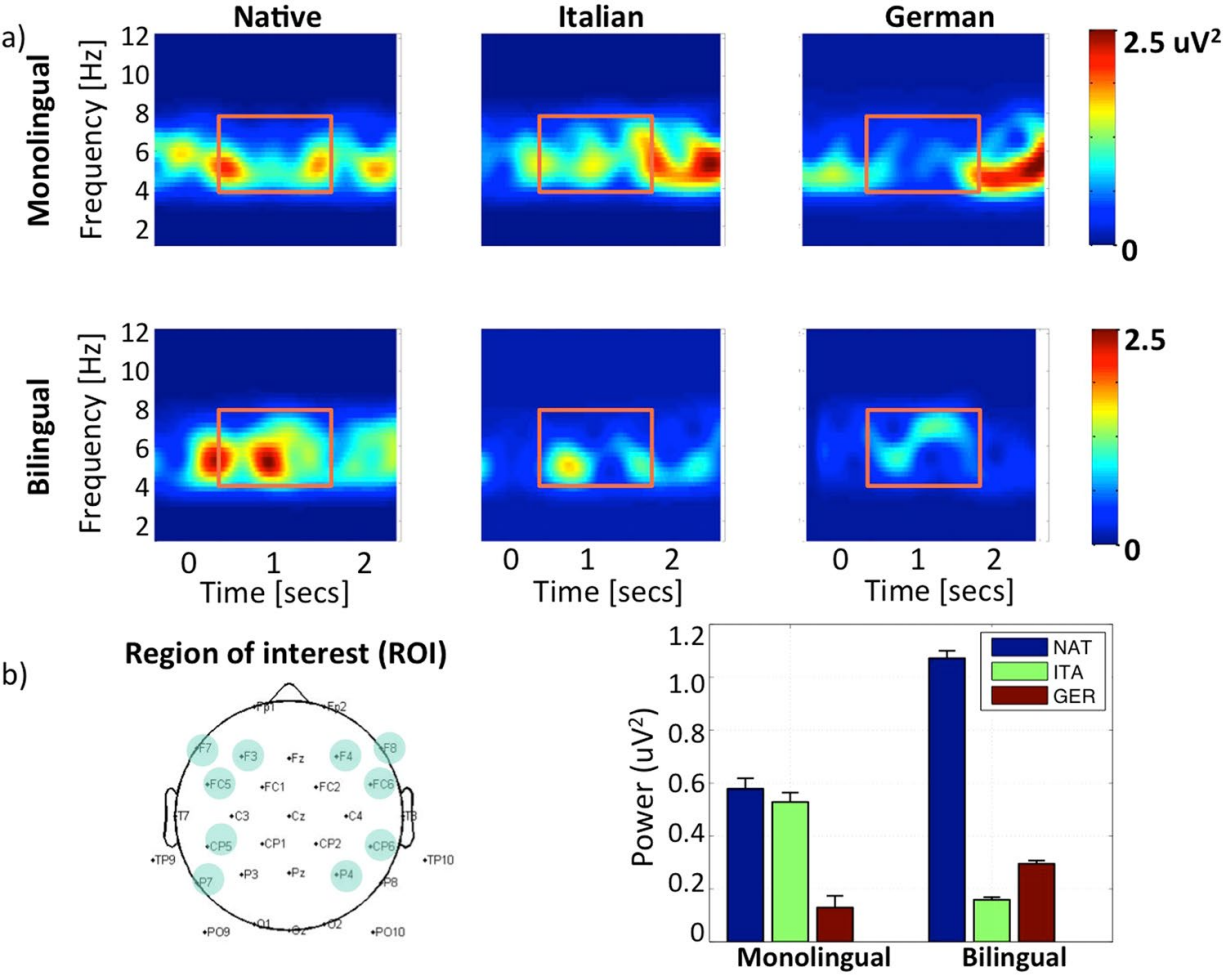
(**a**) Time–frequency power representation of the induced theta response per language and group from 0–12 Hz. For each map, the x-axis represents time (in secs), and the y-axis, frequency (in Hz). Zero corresponds to the onset of the utterances. The color scale codes the variations of power (measured in uV^2^) with respect to a pre-stimulus baseline. The name of the group is written in the left part of the figure and the language on the top of the figure. The orange box highlights the time window of interest (from 400 ms until 1800 ms). (**b**) (Left) EEG electrodes projections of the ROI on the scalp (blue circles). (Right) Mean power for each language in the Theta band from 400 to 1800 ms. The y axis represents the power (uV^2^) from 0 to 1.2 and the x axis represent both the monolingual and the bilingual group. For each group, the mean power is plotted in each bar, the blue bar represents the mean values for the native language, the green for Italian and the red for German. Note: for a better visualization, TFR plots have been preprocessed separately using two filters in the frequency domain: a band pass filter (4–8 Hz) and band stop filter (8–20 Hz).

Planned t-test comparisons only showed significant differences for the Bilingual group when comparing the Native language against German (t(27) = 4.77; p < 0.001) and the Native language versus Italian (t(27) = 2.17; p < 0.001). This comparison confirmed that bilinguals were showing an enhanced Theta activation when listening to their native language (Mean = 1.071, SE = 0.028) as compared to Italian (Mean = 0.159, SE = 0.009) or German (Mean = 0.295, SE = 0.011) (See Fig. 2b).

#### Analysis in the Gamma band range (55–75 Hz)

We ran the same statistical tests as just described for the Gamma band. None of the analyses approached significance. Although we found Gamma activation for each of the languages as compared to the baseline, we did not find interactions or differences between groups (See additional information).

## Discussion and Conclusions

The current study analyzed the brain electrical activity of 4.5-month-old monolingual and bilingual infants to their native language and two foreign languages. Based on previous studies, we differentiated two windows of analysis that allowed us to explore two different responses to the speech signal. The early window of analysis (150–280 ms) was selected to explore the response of familiarity to each language by measuring the latencies of the P200 component. The late window of analysis was selected to analyze language processing reflected in the Theta (400–1800 ms) and Gamma (300–2800 ms) oscillations. We found longer latencies in the P200 component only for the language of the different rhythmic class in monolingual infants. In the late window of analysis, bilinguals showed higher power in the Theta band for the native language as compared with the foreign languages. No differences were observed for the Gamma band.

We hypothesized that in the early window of analysis (150–280 ms), we would observe a P200 component reflecting early discrimination based on familiarity. Although both groups of infants showed a P200 response to all languages, monolinguals showed shorter latencies of the P200 component for the native language compared to the language of the different rhythmic class. The comparison of the P200 responses to Native and Italian did not reach significance. This result parallels the results of Nazzi *et al*.^14^ who reported partial discrimination for within-class language pairs by infants learning American English at 5 months of age. Bilinguals showed no differences in the latency of this component for any of the languages. This lack of differences may reflect bilinguals’ reduced familiarity with each of their two languages. Indeed, because bilinguals’ speech input is split into two languages, they would be less familiar with one of their languages than monolinguals would be with their single native language. Therefore, bilinguals’ P200 response to the native language would be more similar to the one elicited by the unfamiliar languages.

The scarcity of previous research investigating the ERP responses to running speech in young infants makes it difficult to compare our results with previous investigations. Additionally, Peña *et al*.^25^ did not analyze the P200 component as a function of the language infants were listening to, therefore making impossible to determine to which extent our results are in conflict with theirs. A way of testing our hypothesis that language familiarity modulates the latency of the P200 response could be by measuring the correlation between the percentage of exposure to the maternal language and the latency of the P200 component. The design of our study makes the computation of such correlation not without problems, as the distribution of participants as a function of language exposure is very uneven; for instance the vast majority of our monolinguals had virtually no exposure to a non-native language (22 out of the 28 monolingual infants had less than 5% of exposure to a second language). As suggested by a reviewer, we computed the correlation between exposure and the difference between latency to German (a language equally foreign for all infants) and the latency to the native language. The correlation was very modest (r = 0.207, p≈0.10 one-tailed), though in the expected direction. It is worth noticing that such correlation is not different from similar ones involving parental estimate of language input^36^. Future studies where infants’ percentage of exposure to the language of test is properly manipulated may provide further test to our explanation.

Although some of the literature on the P200 component has shown it to appear in central electrodes^27^, we found it in the same topography as Peña *et al*.^25^. One important difference between both studies is the use of short stimuli versus long sentences. It remains to be explored why the topography of the p200 changes when using disyllabic words versus sentences. Finally, Mai *et al*.^27^, found shorter latencies for unfamiliar voices, while in our study we found the opposite familiarity pattern. There are different reasons why this might be the case. First, there were important differences in the stimuli between both studies (a single disyllabic word versus a range of full sentences). It is possible that infants may use different types of information to detect familiarity with so different stimuli, resulting in different patterns of latencies. Second, there were important differences in the age of the participants: 2 months olds in their study versus 4.5 months in ours. In this line, Peña *et al*. (2010) observed that the P200 latency was longer for the younger group of age. Several studies show important changes in the patterns of ERP responses in the first months of life (a well known case is the change in polarity observed in the MMN^23^). At present it is impossible to adjudicate any definitive explanation to the discrepancy observed.

For the late window of analysis, we measured the brain oscillations in the middle Gamma (55–75 Hz) and Theta (4–8 Hz) bands. Previous studies have reported changes in the Theta oscillatory activity of 6-month-old infants related to speech beginning at 400–500 ms after stimulus onset^25,33^. Also, previous studies have reported orientation latencies in response to speech that lasted a mean of 1600 ms in bilinguals^9^. For this reason, we extended the window and analyzed the mean power in the time window beginning at 400 ms until 1800 ms. Following Peña *et al*.^25^, the analysis of the oscillations in the Gamma band was performed over the 300–2800 ms window of analysis and did not show any significant difference. To our knowledge, the only research exploring the neural oscillations of infants in a language discrimination study is Peña *et al*.^25^. These authors found an increase that was exclusive for the native language at 6 but not at 3 months of age in the middle gamma band (55–75 Hz), where they also found increases for languages of the same rhythmic class. The results from the present study may agree with Peña’s *et al*.^25^ study in that at 4.5 months neither the monolingual nor the bilingual group have reached the level of gamma oscillatory maturation needed to reliably detect language discrimination. We attribute our lack of differences to the age of our sample, as oscillatory activity shifts from lower to higher frequencies during infant development^37^ and, crucially, phonological narrowing has not been reported before the second semester of life^31,38^.

We hypothesized that bilinguals would show specific processing of the native speech in the later window of analysis (400–1800 ms), which would reflect an increase in attention to the speech signal to compensate for the reduced amount of exposure to the maternal language. Such additional processing is reflected in the bilingual group having a higher power in the Theta band for the native language as compared to the foreign languages. Although some studies have reported differences in the Theta band in an earlier window of analysis, we did not expect to find any earlier difference due to the properties of our stimuli. Those studies^33,39^ are characterized by the use of short stimuli (syllables) with older infants. Studies using stimuli similar to ours (whole sentences in different languages) have not found changes in Theta Band before 100 ms in adults^28^ or Gamma Band before 300 ms^25^ in 6-month-old infants. Although the comparison of EEG data between infants and adults is not without risks, it is worth noticing that Zion Golumbic *et al*.^40^ related changes in phase of low frequencies, including Theta, to higher attention towards speech in adults. Given the great differences in the attentional system of infants and adults, we cannot claim that they are using the same attentional mechanisms. However, the power modulations that we have observed may be precursors of enhanced attention towards their native language that might be already playing a role. Altogether, the current evidence converges that at 4 months of age bilinguals are already able to pay attention to the prosodic characteristics of their native language. The enhanced attention towards these properties might allow them to identify which of the languages is being spoken at each time.

The primary goal to our research was to better understand the origin of the differences in language processing between monolingual and bilingual infants, already reported at four months of age. We found effects in the early ERP response for monolinguals and changes in the Theta oscillatory activity for bilinguals. Our results mesh well and help explain the origin of Bosch & Sebastian-Galles’^9^ previous behavioral results, showing that, contrary to monolinguals, bilinguals were slower at orienting to the native language than to a foreign one. Such increase in their latencies would reflect bilinguals’ additional processing of the prosodic properties of the familiar language. The reason to favor prosodic processing comes from two different types of evidence. First, as reviewed in the introduction, changes in the Theta band power have been associated to slow-changing properties of the language, in particular, the processing of suprasegmental information (e.g. syllables and prosody)^39^. Second, behavioral studies investigating language discrimination in the 4–5 months age range have provided converging evidence that infants at this age can use prosody to discriminate languages. Bosch & Sebastian-Galles^9^ showed that 4.5-month-old infants can discriminate Catalan and Spanish after low-pass filtering the sentences, a transformation of the speech stream that keeps the prosody and removes detailed phonetic information. Similarly, Nazzi *et al*.^14^ observed that 5-month-old infants rely on prosodic information to distinguish American English from other dialects and languages of the same rhythmic class. Therefore, it is unlikely that bilingual infants in our study were using detailed phonetic information to identify their languages. There is no evidence in the literature that at 4.5 months of age, infants have established representations of their native phonetic repertoire. Vowels are the first phonetic categories to be established and there is no evidence of their existence before 6 months of age^41^. Particularly relevant here are the results of Bosch and Sebastian-Galles^42^ who did not observe evidence of phonetic categories in 4.5 infants exposed to Catalan and Spanish.

The present results also support the hypotheses of enhanced language processing in early bilinguals put forward in studies with audiovisual stimuli. These studies have provided additional evidence for the existence of early specializations to process language in bilinguals. As reviewed in the introduction, Pons, Bosch, & Lewkowicz^21^ observed that already at 4-months of age, bilingual infants pay more attention to the mouth than to the eyes, when compared to same-age monolingual infants, a bias that the authors related to bilinguals’ necessity to keep their languages separated. The same authors observed that this tendency of bilinguals to look to the mouth area when presented with audiovisual dynamic faces extends well beyond the first months of age, and it has also been reported at 8 months of age when looking at dynamic faces even in absence of linguistic stimuli^43^. Also at 8 months of age, studies on visual language discrimination show that only bilinguals keep the capacity of discriminating between visual languages, that is, when seeing faces of people talking in different languages in absence of acoustic information^20,44^.

This work represents the first evidence of differences in the ERP and oscillatory patterns of bilingual and monolingual infant populations in a language discrimination task. Regardless of whether future research will be able to clarify the exact nature of the patterns of results that we have found, our results shed important light in the ways monolinguals and bilinguals perceive their native language. The results of the present research show the earliest evidence of neural adaptations induced by bilingualism.

## Materials and Methods

### Participants

Fifty-six 4-month-old infants participated in this study. All infants were full-term with no reported health problems.

An adapted version of Bosch & Sebastian-Galles’^10^ language exposure questionnaire was administered to establish the infants’ language environment. Twenty-eight infants (14 female) were Catalan (n = 14) or Spanish (n = 14) monolingual (M); while 28 (13 female) were bilingual (B) of both languages exposed predominantly to Catalan (n = 14) or Spanish (n = 14). Mean exposure to the dominant language was 94% (Range 80–100%) in the monolingual group and 63% (Range 50–75%) in the bilingual group. None of the participants were trilingual. Fifty- five additional infants were tested but not included in the final sample due to presenting too many artifacts (26 M; 15 B), crying (5 M; 7 B) or experimenter error (2 B).

Infants’ age range was between 4:00 and 5:00 months. Four months and 18 days was the mean age for the monolingual and 4 months and 14 days for the bilingual group.

The research was conducted in accordance with the principles expressed in the Declaration of Helsinki and approved by the clinical research ethical committee of the IMIM (Hospital del Mar Medical Research Institute), Barcelona (Spain). The infants were recruited from Hospital Quirón and Clínica Sagrada Familia (two private hospitals in Barcelona) and parental informed consent for participation was acquired before running the experiment. All the families received a diploma together with a t-shirt or a bib in appreciation for their voluntary participation.

### Stimuli

For each language, three female native speakers (i.e., three Catalan, three Spanish, three Italian, and three German speakers) were recorded while they were spontaneously explaining what they were seeing in images for children. Speakers were instructed to speak so that the sentences would last around 3 seconds. One native speaker of each language checked that the sentences were correct and sounded native.

We selected 18 utterances per speaker that were equivalent in duration (2,800–3,000 ms) and we normalized the amplitude (see Table 1).

**Table 1:** Mean and Standard Deviation in parenthesis of the duration, number syllables and F0 values per sentence in each of the languages.

	Catalan	Spanish	Italian	German
Mean duration in milliseconds (SD)	2910 (70)	2925 (96)	2920 (70)	2930 (70)
Mean number of syllables (SD)	15.21 (1.95)	16.17(2.32)	17.85 (1.88)	16.09 (2.07)
Range nr of syllables per sentence	10–19	13–23	14–21	11–20
Mean F0 (SD)	221 (11)	207 (17)	205 (17)	196 (16)
Range F0	192–252	181–249	156–231	174–237

### Procedure

Infants were tested at the Laboratori de Recerca en Infància at Universitat Pompeu Fabra (Barcelona, Spain). The procedure was similar to the one used by Peña *et al*.’s^25^ in which infants were seated on their parents’ lap at a 60 cm distance from the 19-inch screen and watched infant-appropriated images while the sentences were played through loudspeakers placed right behind the screen. Brain electrical activity (EEG) was recorded during passive listening to utterances in Catalan or Spanish (depending on the native or predominant native language of the infant), Italian (unknown foreign, same rhythmic class), and German (unknown foreign, different rhythmic class) languages.

A total number of 156 utterances (52 utterances per language) were selected. None of the sentences was repeated during the experiment. Following Peña *et al*.’s^25^ design, utterances were pseudo-randomly presented in blocks composed of four consecutive utterances per language. Two consecutive utterances from the same speaker or two consecutive blocks from the same language were never presented. Within each block, utterances were separated by 800-ms silent pauses and between block silent pauses lasted between 2,000–2,200 ms. The order of presentation was randomized for each participant. The experiment was paused if the infant manifested discomfort or fussiness and the study was stopped if the infant started crying.

### Electrophysiological recording and EEG processing

EEG signal was recorded with Ag-AgCl active electrodes from 32 scalp locations of the 10–20 system, referenced to the vertex. The signal was amplified using a BrainAmp amplifier (Brain Products GmbH, Munich, Germany) with a bandpass filter of 0.1–100 Hz, digitized at a sampling rate of 500 Hz. Then a notch filter of 50 Hz was applied offline. The raw EEG signal was off-line averaged and re-referenced to all channels. Triggers were time-locked to stimulus (sentence) onset. The averaging epochs were taken from 800 ms prior to trigger on set to 3000 ms and a baseline correction was first applied for the pre-stimulus range from −100 to 0 ms. Trials inspection was done visually in three steps: (a) displaying one trial at a time, that means, plotting all trials according to the maximum amplitude over all channels, (b) plotting all trials from one channel at a time, and (c) checking all trials by variance and z-values, that means, trials with very high variance or z-values were rejected. Epochs containing artifacts caused by eye movement were removed using an Independent Component Analysis (ICA). Artifacts were visually removed by independent components (ICs) and their projections on topographic scalp maps^43^. Channels highly contaminated by noise (for example, peripheral electrodes) were selected for a deeper analysis such as single time-frequency inspection, and finally, channels selected as bad channels were extrapolated just with good channels (neighbors). We used a minimum number of three good channels as neighbors. Only participants at least with 10 valid utterances per language (both in ERPs and also in time-frequency) were retained for analysis.

#### ERP analysis

Following Peña *et al*., four electrodes (F3, FC5, F4, and FC6) localized on the frontocentral region defined our Region Of Interest (ROI). P200 component is often distributed around this area of the scalp^25^ (see Fig. 1). We performed peak amplitude and latency detection in the 150–280 ms window and for each participant measured the latency of the first positive peak of the window. Statistical analysis was performed using three different tests. First, a repeated measures ANOVA with a between factor, Group (Monolingual, Bilingual) and a within factor, language (Native, Italian, German) was run. The time-windows of interest and the ROI were selected a priori based on previous literature^25,28^. Next, each group was separately compared by a one-way-ANOVA with Language as factor, and finally, a planned paired t-test was applied for three comparisons: Native vs Italian, Native vs German and Italian vs German. All the analysis were performed separately for the median values of amplitudes and latencies, which is the middle score within a data set and is the least affected by outliers.

#### Time-frequency analysis

The FieldTrip Matlab^©^ (Version: 8.0, MathWorks V.R2012b, Natick, MA) package was used for data analysis^45^. For the brain oscillations, we calculated Theta and Gamma power separately: (4–8 Hz; frequency step, 0.2 Hz) for Theta and (20–80 Hz; frequency step,1 Hz) for Gamma. To obtain power spectral estimates in the frequency domain, Fast Fourier Transformation were used for each epoch. Spectral power estimates were then averaged within the Theta (4–8 Hz) and Middle Gamma (55–75 Hz) ranges. Ten electrodes (FC6, CP6, P4, F4, F8, F3, FC5, CP5, P7, F7) localized on the fronto-temporo-parietal regions (see Fig. 2b) defined our Region Of Interest (ROI).

Time-frequency decomposition of all EEG segments was calculated using Hanning tappers with length equal to 900 ms (Theta band) and 300 ms (Middle Gamma band). The time-frequency transformed data were then normalized against an 800-ms prestimulus baseline and averaged across (non-contaminated) epochs in the ROI for each infant and for each language. Thus, estimates of signal power contained induced components to stimulus onset^46^. Normalization consisted in subtracting the power of the baseline from the power of the utterance. We visually inspected the time-frequency plots (TFRs) for each group and language^47^ (see Fig. 2a). TFRs highlight the power changes of the signal relative with the stimulus. As for the ERPs, similar tests were used in the statistical analysis. However, being the TFRs bi-dimensional signals, for each comparison we extracted the median power of the windows of interest, and submitted for statistical analysis^25,28^.

### Data availability

The datasets and scripts generated during the current study are available in the GITHUB repository, https://github.com/CGuerrero13/Nacar.et.al.2017.Scientific.Reports.

## Electronic supplementary material

Supplementary information.
