# Supplementary material for: Evoked and oscillatory EEG activity differentiates language discrimination in young monolingual and bilingual infants
Source: Sci Rep. 2018 Feb 9;8:2770. doi: 10.1038/s41598-018-20824-0 (PMC5807452; doi:10.1038/s41598-018-20824-0)

Evoked and oscillatory EEG activity differentiates language discrimination in young monolingual and bilingual infants

Loreto Nacar Garcia[1*], Carlos Guerrero-Mosquera[2], Marc Colomer[2], Nuria Sebastian-Galles[2]

[1]Infant Studies Centre, University of British Columbia, 2136 West Mall, Vancouver, BC, V6T 1Z4, Canada
[2]Center for Brain and Cognition, Universitat Pompeu Fabra, Ramon Trias Fargas, 25-27
08005 Barcelona, Spain
*Correspondence to loreto.nacar@psych.ubc.ca

**ANOVA**	F value	p value	SS effect	SS total	eta squared
**P200**					
**RM_ANOVA**					
Group	0.668	0.4213	0.001	0.167	0.006
Language	2.209	0.120	0.003	0.167	0.018
Group*Language	4.110	0.022	0.006	0.167	0.036
**One-Way-ANOVA**					
Monolingual	3.886	0.024	0.008	0.094	0.088
Bilingual	0.672	0.513	0.001	0.073	0.017
**Theta**					
**RM_ANOVA**					
Group	0.120	0.731	0.127	104.732	0.001
Language	2.852	0.066	2.351	104.732	0.022
Group*Language	5.948	0.004	21.428	104.732	0.205
**One-Way-ANOVA**					
Monolingual	0.558	0.574	1.117	82.132	0.014
Bilingual	15.208	<0.001	6.135	22.437	0.273

**T-Test**	t value	p value	Cohen’s d	effect-size r_{YI}
**P200**				
**Monolingual**				
Native-Italian	-0.035	0.971	-0.019	0.009
Native-German	-2.891	0.007	-1.604	0.626
Italian-German	-2.501	0.018	-1.387	0.569
**Bilingual**				
Native-Italian	-1.277	0.212	-0.708	0.334
Native-German	-0.056	0.955	-0.031	0.016
Italian-German	0.950	0.350	0.527	0.255
**Theta**				
**Monolingual**				
Native-Italian	-1.032	0.310	-0.572	0.275
Native-German	0.047	0.962	0.026	0.013
Italian-German	1.198	0.241	0.665	0.315
**Bilingual**				
Native-Italian	4.387	<0.001	2.43	0.77
Native-German	5.169	<0.001	2.867	0.820
Italian-German	-0.309	0.759	-0.171	0.085

STAT analysis: ERP amplitudes

Ttest within (p-values):

	NAT-ITA	NAT-GER	ITA-GER
**MONO**	0.539	0.339	0.785
**BI**	0.318	0.688	0.234

Ttest between (p-values):

NAT	GER	ITA
0.483	0.753	0.524

ANOVA
Monolingual: n.s. (p= 0.69)
Bilingual: n.s (p=0.49)

Repeated Measures ANOVA
Group: n.s. (p= 0.83)
Language: n.s (p=0.56)
GroupxLang: n.s (p=0.60)

MONO

	Mean	Median	Sigma
NAT	0.455	0.418	0.414
ITA	0.398	0.371	0.344
GER	0.376	0.362	0.305

BI

	Mean	Median	Sigma
NAT	0.382	0.426	0.362
ITA	0.453	0.415	0.303
GER	0.348	0.385	0.349

Groups:

	Mean	Median	Sigma
MONO	0.410	0.365	0.354
BI	0.394	0.397	0.338

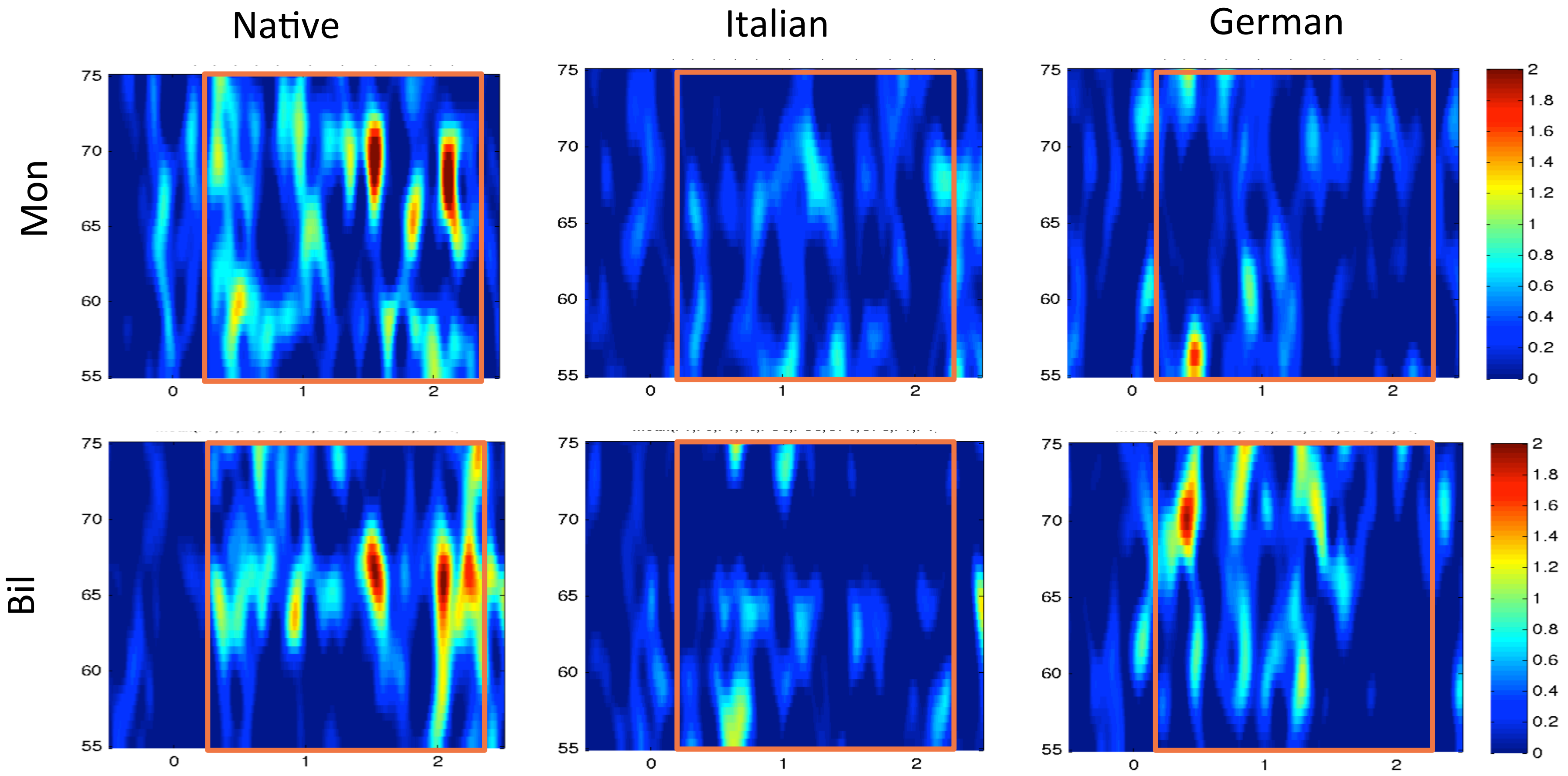

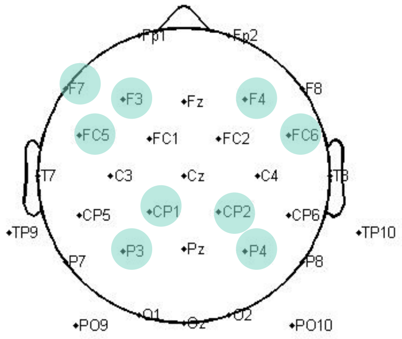

Time intervals: 0.3-2.8 secs; 0.4-1.85secs
Freq sub-bands: 55-75Hz;65-75Hz
Stat results (red box interval):
n.s (see next slides for more details)

Statistics between 55-75Hz and 0.3 -2.8secs.

MONO

	Mean	Median	Sigma
NAT	1.025	1.023	0.273
ITA	1.148	1.051	0.583
GER	1.195	1.093	0.380

BI

	Mean	Median	Sigma
NAT	1.269	1.048	1.121
ITA	0.996	0.953	0.301
GER	1.085	1.001	0.015

Groups

	Mean	Median	Sigma
MONO	1.123	1.051	0.432
BI	1.117	1.003	0.733

STAT analysis resume (55-75Hz,0.3-2.8 secs)

Ttest within (p-values):

	NAT-ITA	NAT-GER	ITA-GER
**MONO**	0.35	0.05	0.57
**BI**	0.26	0.43	0.49

Ttest between (p-values):

NAT	GER	ITA
0.27	0.37	0.23

ANOVA
Monolingual: n.s. (p= 0.32)
Bilingual: n.s (p=0.37)

RM ANOVA
Group: n.s. (p= 0.95)
Language: n.s (p=0.76)
Group x Language: n.s (p=0.16)

Gamma power

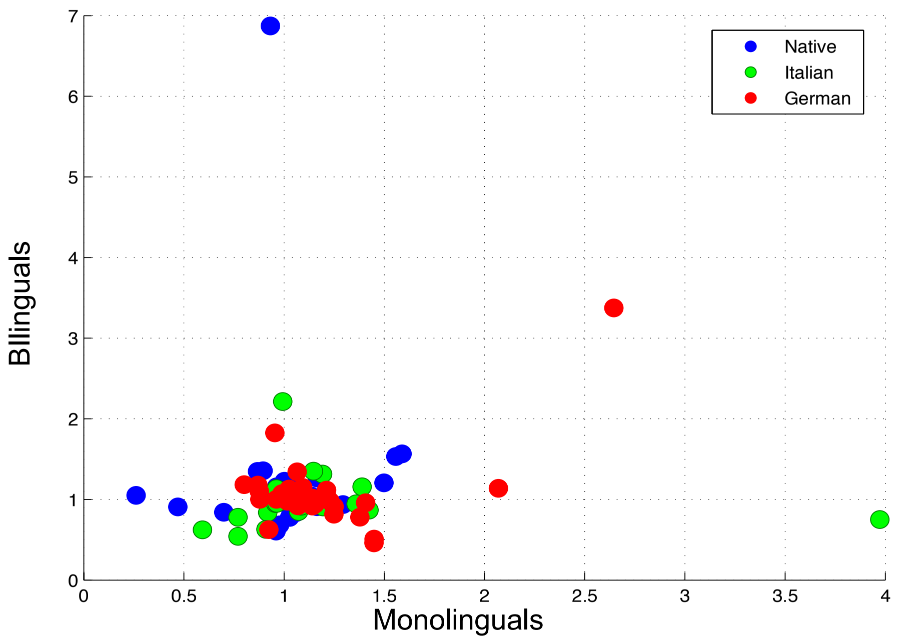

Categorical scatter plot

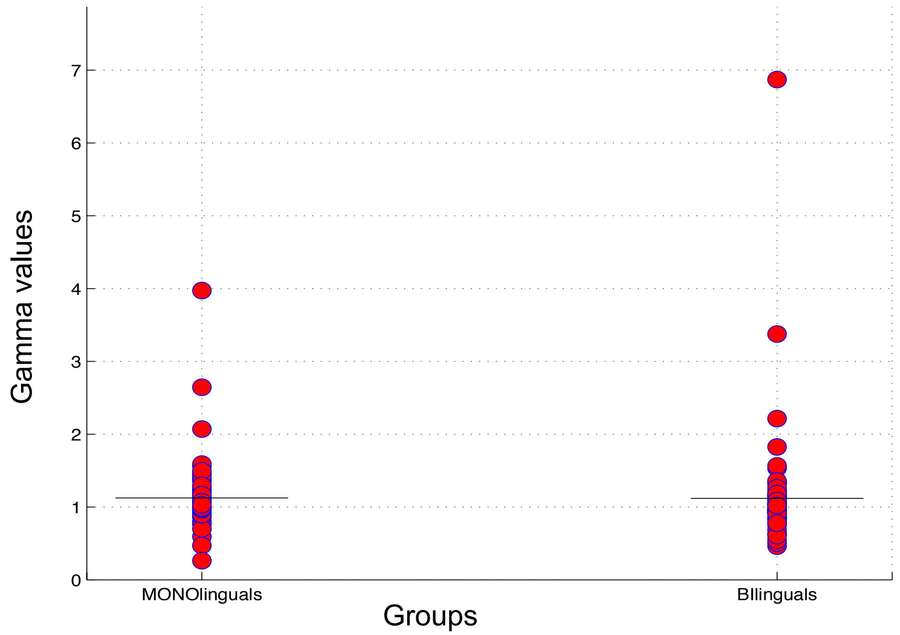

Supplement: Supplementary file 1 — Supplementary information. [file 41598_2018_20824_MOESM1_ESM.pdf]
